# Supplementary material for: Increasing tropical cyclone intensity in the western North Pacific partly driven by warming Tibetan Plateau
Source: Nat Commun. 2024 Jan 5;15:310. doi: 10.1038/s41467-023-44403-8 (PMC10770404; doi:10.1038/s41467-023-44403-8)
Supplement: Supplementary file 1 — Supplementary Information [file 41467_2023_44403_MOESM1_ESM.pdf]

Increasing tropical cyclone intensity in the western North Pacific partly driven by warming Tibetan Plateau

Jing Xu[1], Ping Zhao[1, 2*], Johnny C. L. Chan[3,4], Mingyuan Shi[5], Chi Yang[6], Siyu Zhao[7], Ying Xu[8], Junming Chen[1], Ling Du[9], Jie Wu[10], Jiaxin Ye[1], Rui Xing[11], Huimei Wang[1], Lu Liu[1]

[1]Chinese Academy of Meteorological Sciences, Beijing 100081, China;

[2]Plateau Atmosphere and Environment Key Laboratory of Sichuan Province, College of Atmospheric Science, Chengdu University of Information Technology, Chengdu 610225, China;

[3]Guy Carpenter Asia-Pacific Climate Impact Centre, School of Energy and Environment, City University of Hong Kong, Hong Kong, China;

[4]Asia-Pacific Typhoon Collaborative Research Center, Shanghai 201306, China;

[5]National Meteorological Information Center, Beijing 100081, China;

[6]Faculty of Geographical Science, Beijing Normal University, Beijing 100875, China;

[7]Department of Atmospheric and Oceanic Sciences, University of California, Los Angeles, Los Angeles, USA;

[8]National Climate Center, Beijing 100081, China;

[9]Frontier Science Center for Deep Ocean Multispheres and Earth System (FDOMES) and Physical Oceanography Laboratory; and College of Oceanic and Atmospheric Sciences, Ocean University of China, Qingdao 266100, China;

[10]Gannan Normal University, Ganzhou 341000, China;

[11]Meteorological Service in Binhai New Area, Tianjin 300457, China.

Nature Communications

Corresponding author: Ping Zhao (zhaop@cma.gov.cn)

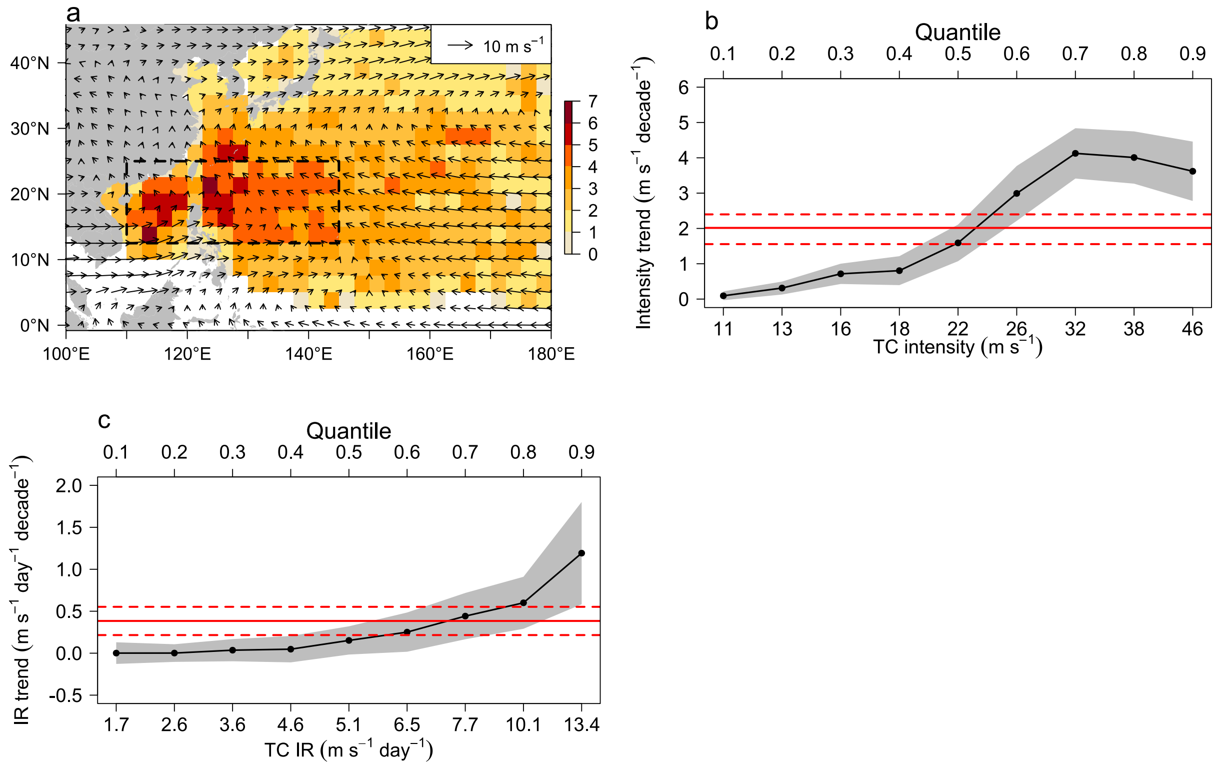

Supplementary Figure 1. Climatological characteristics of observed tropical cyclone (TC) activity, intensity, and intensification rate. a The distributions of the number of the climatological mean TC records (color) and 850-hPa wind (m s^{-1}; vector) during 1988–2018, in which the dashed box is for the monsoon trough (MT) area. **b** Linear trends (m s^{-1} decade^{-1}) in different quantiles of TC intensity (m s^{-1}) in the MT area and their point-wise confidence bands at the 0.05 level (shaded), in which the red solid line indicates the linear trend in TC intensity for all cases in the MT area and the red dashed lines are the linear trends for the point-wise confidence band at the 0.05 level. **c** As in **b** but for the TC intensification rate (m s^{-1} day^{-1} decade^{-1}). All data are for July-August-September (JAS). This figure was created using R version 4.3.2 (https://www.r-project.org/). Source data are provided as a Source Data file.

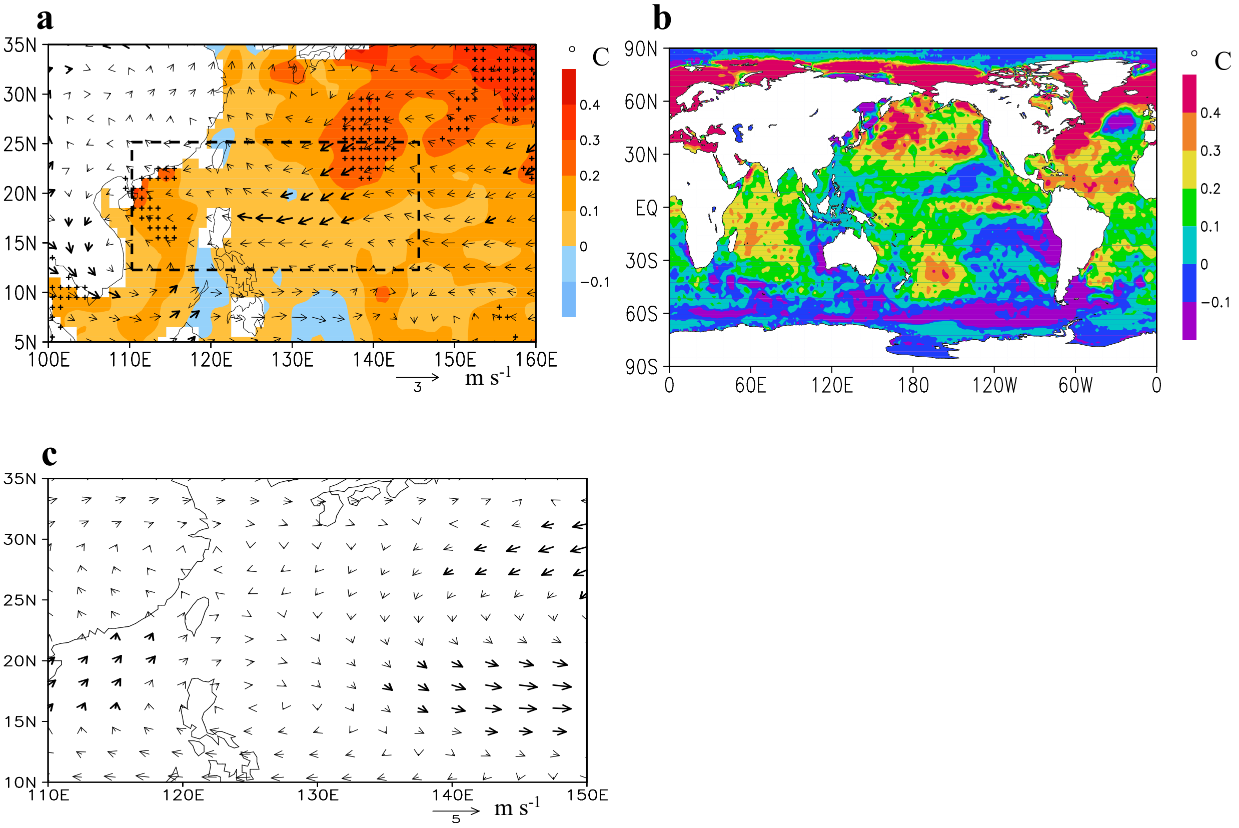

Supplementary Figure 2. Anomalies of observed surface sea temperature (SST) and wind and forced vertical wind shear (VWS) by global SST change. **a** Differences of SST (shaded; °C) and 850 hPa wind (vectors; m s^{-1}) between P2 (period 2002-2018) and P1 (period 1988-2001), in which the plus sign is significant at the 0.05 confidence level and thick vectors are at the 0.05 level. **b** Differences of SST (shaded; °C) between P2 and P1. **c** Differences in VWS (m s^{-1}) forced by the global SST change between P2 and P1 (thick vectors are at the 0.05 level). All data are for JAS. This figure was created using Grid Analysis and Display System (GRADS) Version 2.0.a9.oga.1 (https://sourceforge.net/projects/opengrads/files/). Source data are provided as a Source Data file.

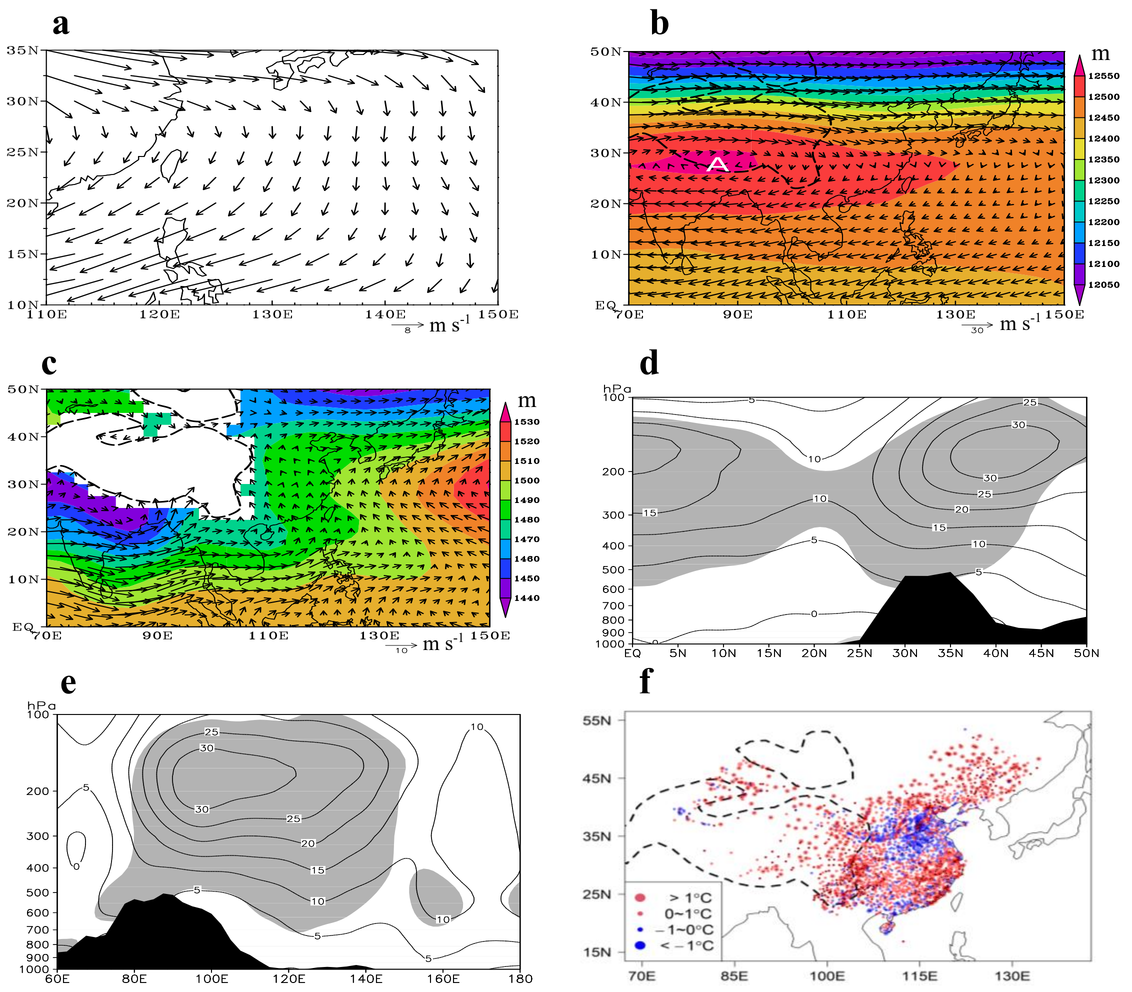

Supplementary Figure 3. Atmospheric characteristics and anomalies between P2 and P1. a Climatological mean VWS (m s^{-1}) between 200 hPa and 850 hPa. **b** Climatological mean 200-hPa geopotential height (m; shaded) and wind (m s^{-1}; vector). **c** As in **b** but for 850 hPa. **d** Latitude-height section of differences in geopotential height (m; light shaded areas are at the 0.05 level and the black shaded area indicates the topography) between P2 and P1 along 80°-100°E. **e** Same as in **d** but along 30°-40°N. **f** Differences in surface air temperature (SAT) at meteorological stations (ºC; black dots are at the 0.05 level) between P2 and P1. All data are for JAS. **a-c** were created using Grid Analysis and Display System (GrADS) Version 2.2.1.oga.1 (https://sourceforge.net/projects/opengrads/files/grads2/2.2.1.oga.1/). **d-e** were created using

Grid Analysis and Display System (GRADS) Version 2.0.a9.oga.1 (https://sourceforge.net/projects/opengrads/files/). **f** was created using R version 4.3.2 (https://www.r-project.org/). Source data are provided as a Source Data file.

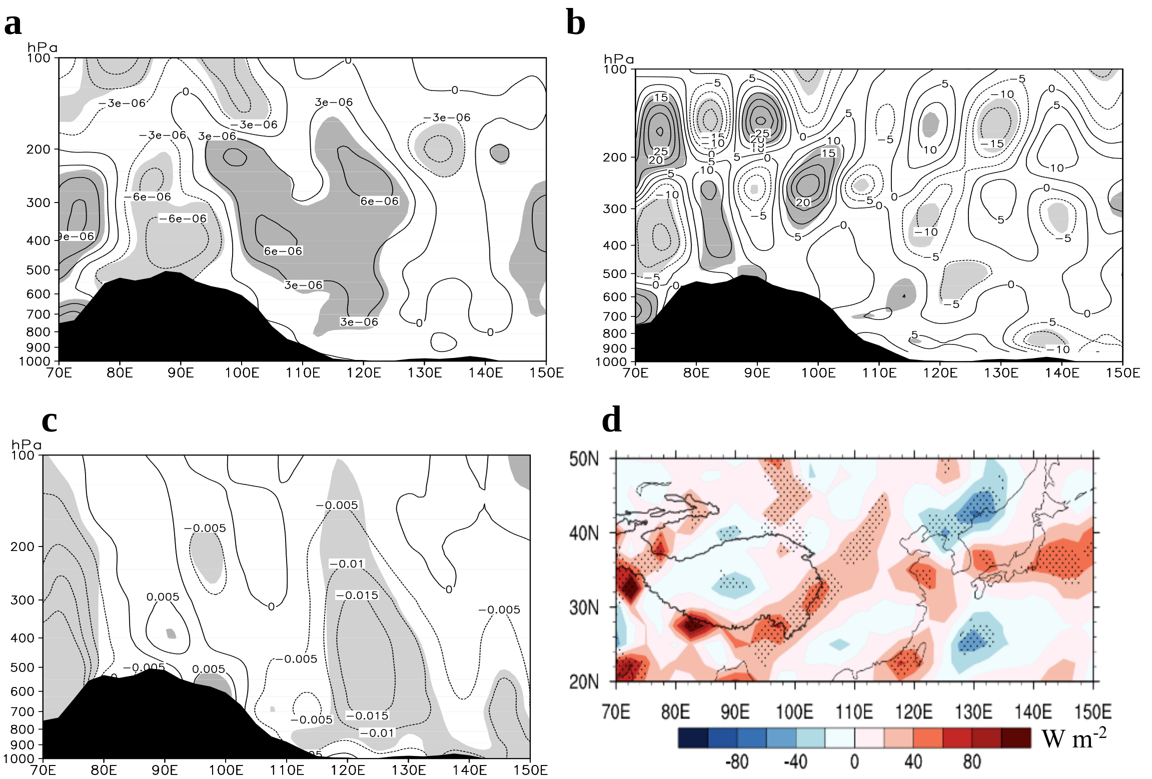

Supplementary Figure 4. Dynamic and thermodynamic anomalies of observed atmospheric circulation between P2 and P1. a Longitude-height section of differences in temperature advection ($-\boldsymbol{V} \cdot \nabla T$; K s^{-1}) along 35°N, in which shaded areas are at the 0.05 level and black shaded area indicates the topography. **b** Same as in **a** but for $\frac{\partial}{\partial z}(\boldsymbol{V} \cdot \nabla\zeta + \beta v)$ ($\times10^{-15}$ $m^{-1}s^{-1}$). **c** Same as in **a** but for *p*-vertical velocity (Pa s^{-1}). **d** Differences in $\langle Q_2 \rangle$ (W m^{-2}), in which dots are at the 0.05 level. All data are for JAS. **a-c** were created using Grid Analysis and Display System (GRADS) Version 2.0.a9.oga.1 (https://sourceforge.net/projects/opengrads/files/). **d** was created using NCAR Command Language (NCL) Version 6.5.0 (https://www.ncl.ucar.edu/Download/). Source data are provided as a Source Data file.

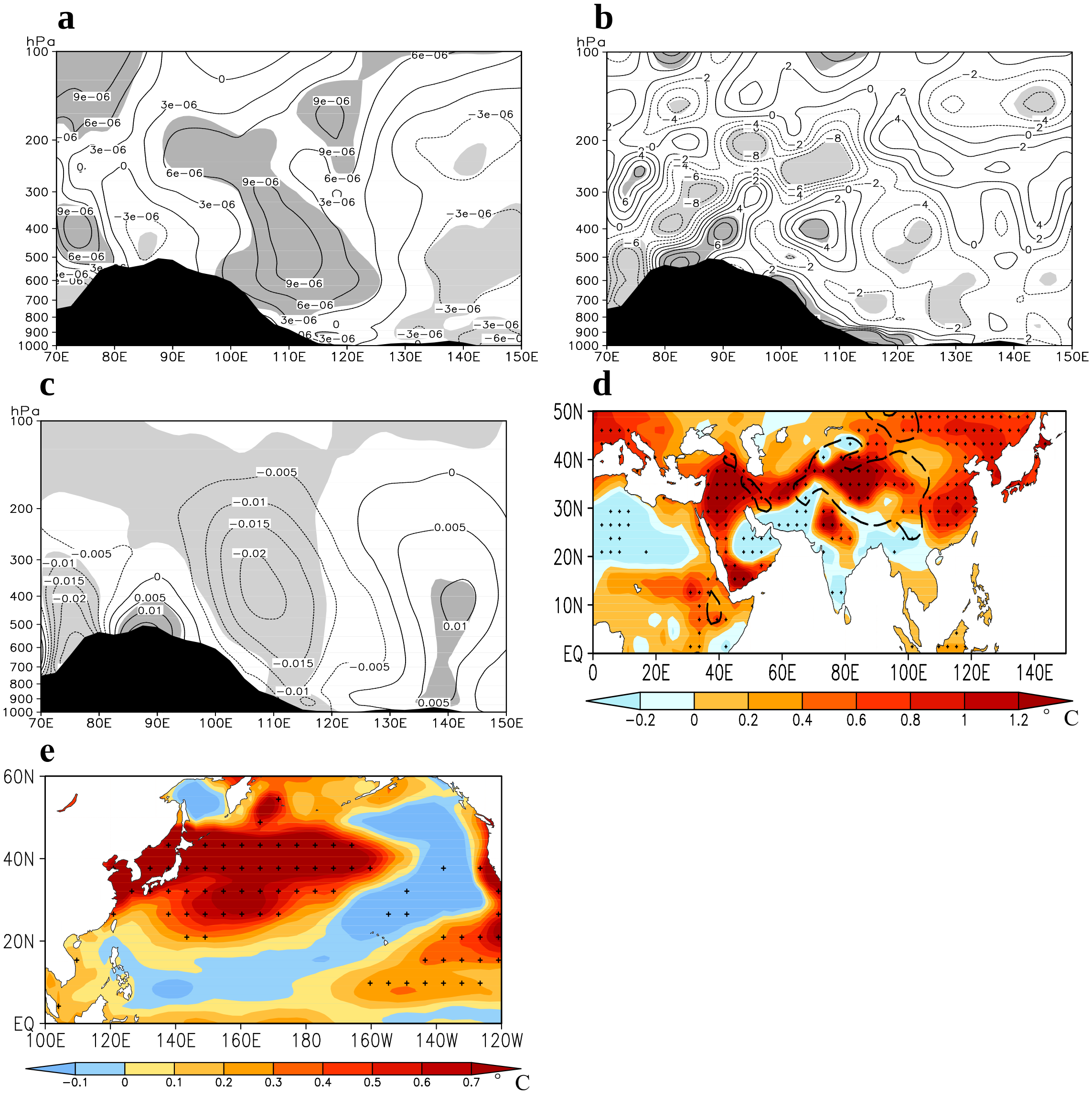

Supplementary Figure 5. Anomalies forced by the warming TP. a For longitude-height section of temperature advection (-$\boldsymbol{V}\cdot\nabla T$; K s^{-1}) along 40°N, in which shaded areas are at the 0.05 level. **b** Same as in **a** but for $\frac{\partial}{\partial z}(\boldsymbol{V}\cdot\nabla\zeta+\beta v)$ (×10^{-15} m^{-1}s^{-1}). **c** Same as in **a** but for *p*-vertical velocity (Pa s^{-1}). **d** For SAT (°C; shaded), in which the plus signs are at the 0.05 level. **e** For SST. All data are for JAS. This figure was created using Grid Analysis and Display System (GRADS) Version 2.0.a9.oga.1 (https://sourceforge.net/projects/opengrads/files/). Source data are provided as a Source Data file.

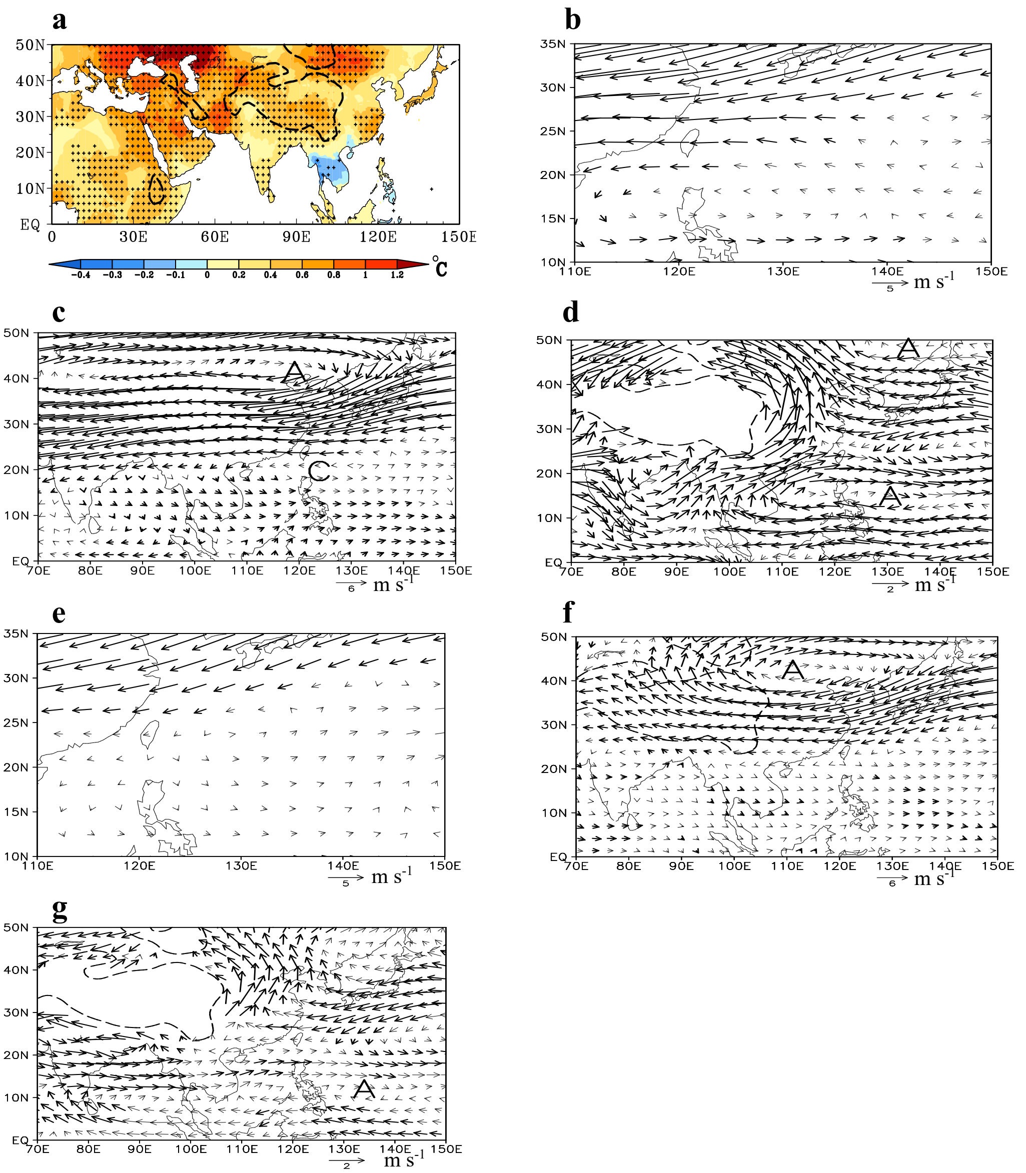

Supplementary Figure 6. Observed and forced SAT, wind, and VWS anomalies. a Differences in observed SAT (°C; shaded; the plus sign is at the 0.05 level) between P2 and P1. **b** Differences in VWS (m s^{-1}) between 200 hPa and 850 hPa forced by warming Asia relative to the control, in which thick vectors are at the 0.05 level. **c** Same as in **b** but for 200-hPa wind. **d** Same as in **b** but for 850-hPa wind. **e** Same as in **b** but for warming Europe. **f** Same as in **e** but for 200-hPa wind. **g** Same as in **e** but for 850-hPa wind. All data are for JAS. This figure was created using Grid Analysis and Display System (GRADS) Version 2.0.a9.oga.1 (https://sourceforge.net/projects/opengrads/files/). Source data are provided as a Source Data file.

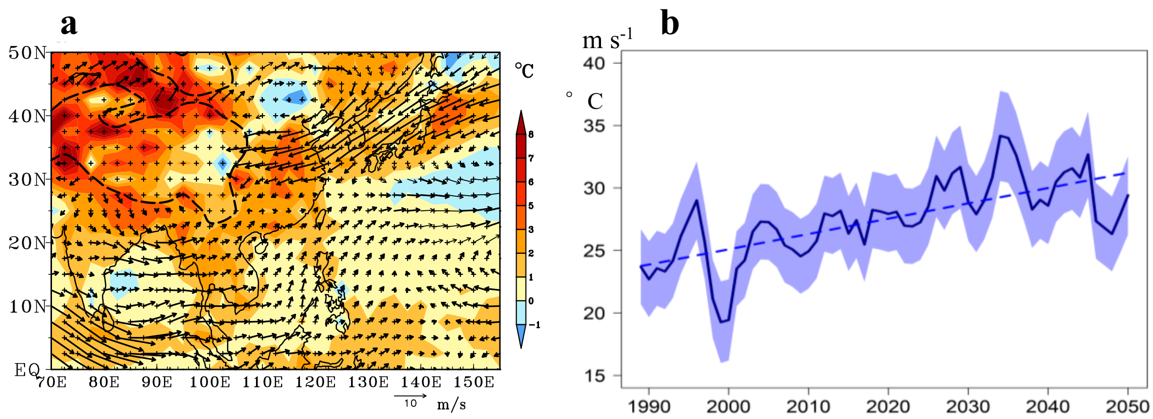

Supplementary Figure 7. Projections of SAT, VWS, and TC intensity by the CMIP 5 NCAR Community Climate System Model version 4 (CCSM4) under the Representative Concentration Pathway (RCP) 4.5 scenario. a Differences of JAS SAT (°C; shaded; the plus sign is significant at the 0.05 level) and VWS (m s^{-1}; vectors; black vectors are at the 0.05 level) between 2022–2050 and 1988-2018. **b** The fitted JAS TC intensity (m s^{-1}; the solid line) with the three-year running mean VWS time series, in which the projection is under the RCP 4.5 scenario, with their interquartile ranges at the 0.05 level, and the dashed line is for the linear trend. **a** was created using Grid Analysis and Display System (GrADS) Version 2.2.1.oga.1 (https://sourceforge.net/projects/opengrads/files/grads2/2.2.1.oga.1/). **b** was created using R version 4.3.2 (https://www.r-project.org/). Source data are provided as a Source Data file.

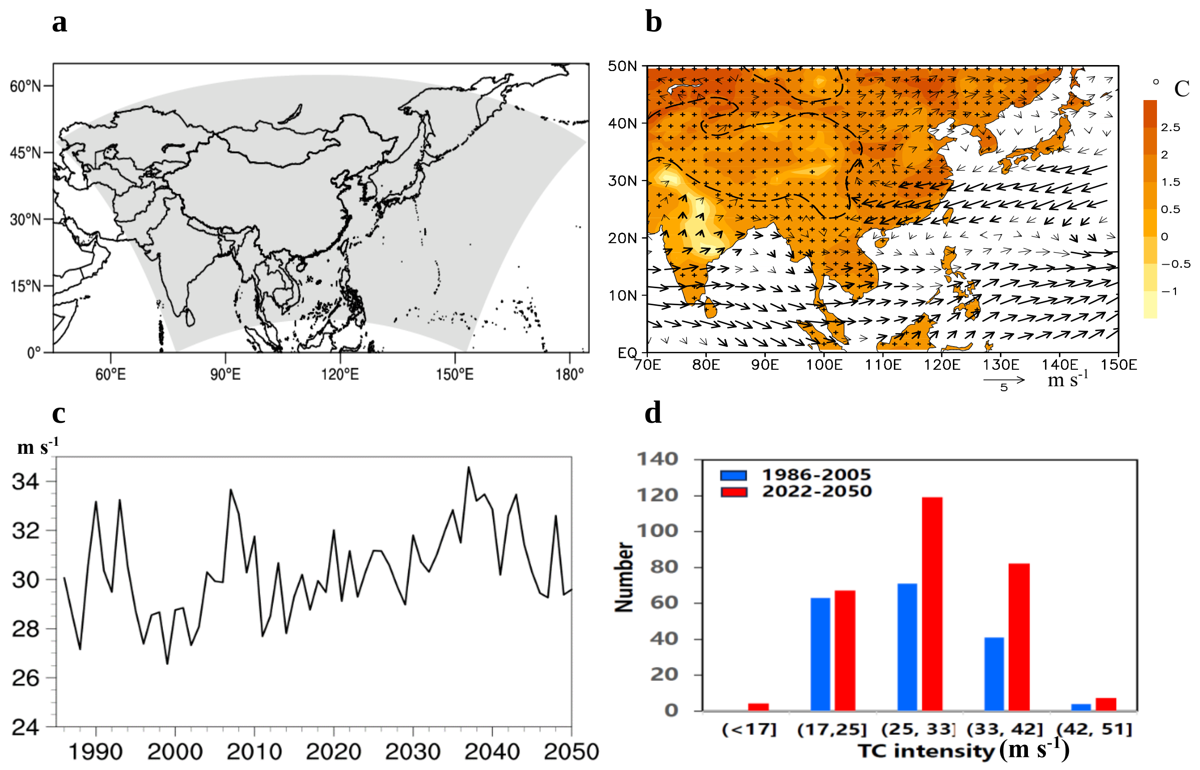

Fig. S8 The model domain and projections of atmospheric circulation and TC intensity. a The CORDEX-East Asia domain (shaded). **b** Differences of the CMIP5 HadGEM2-ES projected (2022-2050) surface temperature (°C; shaded) and VWS between 200 hPa and 850 hPa (m s^{-1}; vector) under the RCP4.5 scenario relative to the reference period (1986-2005), in which the plus sign is significant at the 0.05 level for temperature and thick vectors are at the 0.05 level. **c** The temporal curve of the RegCM4 maximum 10-m wind velocity (m s^{-1}) around the TC center averaging over 110°E-145°E, 15.5°N-27.5°N during 1986-2050, in which 1986-2005 is the historical simulation and 2006-2050 is under the RCP4.5 scenario. **d** Relationship between the RegCM4 TC number and intensity in 110°E-145°E, 15.5°N-27.5°N. All data are for JAS. **b** was created using Grid Analysis and Display System (GRADS) Version 2.0.a9.oga.1 (https://sourceforge.net/projects/opengrads/files/). **c** was created using NCAR Command Language (NCL; http://www.ncl.ucar.edu/) 6.3.0. **d** was created using Microsoft Excel. Source data are provided as a Source Data file.

Supplementary Table 1. Correlation of JAS TC intensity with OHC, SST, MPI, VWS, and TP surface pressure (TPSP) during 1988-2018, in which the asterisk sign is significant at the 0.05 level. Source data are provided as a Source Data file.

TC	OHC	SST	MPI	VWS	TPSP
Original series	-0.33	-0.39*	-0.3	-0.54*	0.28
Detrended series	-0.69*	-0.58*	-0.5*	-0.48*	0.03
Smoothed series	0.15	0.18	0.24	-0.85*	0.64*
